# Clinical guidelines—the challenges and opportunities: What we have learned from the case of misoprostol for postpartum hemorrhage

**DOI:** 10.1002/ijgo.12704

**Published:** 2018-11-21

**Authors:** Jessica L. Morris, Samia Khatun

**Affiliations:** ^1^ International Federation of Gynecology and Obstetrics (FIGO) London UK

**Keywords:** Misoprostol, Postpartum hemorrhage, PPH, PPH guidelines

## Abstract

**Objective:**

To establish whether national guidelines for postpartum hemorrhage (PPH) reflect new scientific evidence on misoprostol, and determine the challenges faced in their implementation.

**Methods:**

A web‐based survey was sent by email to 130 national societies of obstetrics and gynecology (FIGO Member Associations) in 2016. The survey, composed of 18 questions, covered national guidelines on PPH with particular reference to misoprostol, the creation of national guidelines, and challenges to implementation.

**Results:**

Completed surveys were received from 69 societies, for a 53% response rate. The key findings were that many countries lacked comprehensive, up‐to‐date, evidence‐based national guidelines providing guidance on misoprostol use; recommended regimens were very different in the national guidelines as well as between international and regional guidelines that are most often used as referencing documents; and there are a variety of challenges to implementation of guidelines.

**Conclusion:**

There is a need, especially in countries with high maternal mortality, to establish mechanisms that ensure the existence of up‐to‐date, comprehensive, evidence‐based guidelines on PPH. This can be difficult given conflicting guidance at the international level. Regional and international societies should prioritize clinical updates and ensure their dissemination and implementation.

## Abstract 2

A survey of 69 national societies of obstetrics and gynecology showed that many lacked comprehensive, up‐to‐date, evidence‐based national guidelines on postpartum hemorrhage, including misoprostol use.

## 1 INTRODUCTION

Every day, approximately 830 women die from preventable causes related to pregnancy and childbirth.^1^ Almost all of these deaths occur in low‐resource settings, disproportionately affecting women living in rural areas and among poorer communities.^2^ Between 1990 and 2015, the number of maternal deaths worldwide dropped by 44%, with some regions making greater headway than others.^1^ The Sustainable Development Goal (SDG) target for 2016–2030 is to reduce the global maternal mortality ratio to less than 70 per 100 000 live deliveries, with no country having a maternal mortality rate of more than twice the global average.^1^

Hemorrhage is the leading direct cause of maternal mortality, accounting for 27.1% of maternal deaths worldwide. More than two‐thirds of reported hemorrhage deaths were classified as postpartum hemorrhage (PPH).^3^ Uterine atony is the cause of the majority of PPH cases. Administration of uterotonics is an important component in the prevention and treatment of PPH once the cause has been established as uterine atony. The gold‐standard uterotonic for management of PPH due to uterine atony is oxytocin delivered by intravenous or intramuscular injection. Where oxytocin is not available, storage conditions are inadequate, or staff are not trained to administer it safely, misoprostol (available in tablet form) is the current best alternative given that it is a simple and inexpensive product that is both light and heat stable.^4^, ^5^

Owing to the global imperatives to reduce maternal mortality, and given the existing knowledge base, international recommendations specify that the following aspects must be ensured in high‐risk countries: uterotonic availability; medicines are listed on international (WHO) and national essential medicines lists (EML) in correct dosages; and international and national guidelines on PPH management are in place that support the provision of these uterotonics, and that the guidelines are fully utilized.^6^

Ensuring the existence and implementation of evidence‐based clinical guidelines is a key objective for many focus areas of global health concern. Regarding maternal health and PPH, evidence shows that it is an effective component. A recent systematic review evaluated the impact of new or updated guidelines for PPH management implemented in clinical practice. In four of seven trials in the review, the numbers of PPH cases declined after the intervention.^7^

While an array of international clinical guidelines exist on the prevention and treatment of PPH, little is known about whether these guidelines are utilized, whether they are translated into national guidelines, or used to get medicines listed on national EMLs. The aim of the present study, arising from FIGO's interest in the use of international guidelines, was to understand whether new scientific evidence is incorporated into national clinical guidelines and EMLs—important steps toward improving maternal health.

## 2 MATERIALS AND METHODS

A web‐based survey was developed and sent by email to 130 FIGO Member Associations (MAs) on February 5, 2016. Consent was requested in the email and at the start of the survey. Three email reminders were sent (February 15, February 29, and March 7) before the survey was closed on March 21, 2016. The survey comprised 18 questions, with both single response and multiple response questions. The broad question areas covered were: national guidelines on PPH and their content with regard to misoprostol, the creation of national guidelines, challenges to their implementation, and the inclusion of misoprostol on national EMLs. Data were imported into Excel (Microsoft, Redmond, WA, USA) where basic analysis was conducted.

We decided to ask questions specifically about misoprostol for three reasons. Firstly, misoprostol may be more practical in the absence of oxytocin owing to the reasons noted above, making it an important component of an integrated package of PPH interventions, especially in low‐resource and community settings. Secondly, misoprostol—a relatively new drug supported by new science—can be used as an indicator for how quickly guidelines respond to the latest evidence on PPH management. Both FIGO and WHO produced guidelines for prevention and treatment of PPH in 2012 (in 2017 FIGO produced an updated misoprostol only recommended dosage chart, but no changes were made to the recommended dosages for use of misoprostol for PPH management); therefore, misoprostol can serve as an interesting point of comparison for both international and national recommendations. Furthermore, in 2011 and 2015, misoprostol was included in the WHO EML for use in PPH prevention and management, respectively. Thirdly, since FIGO has been involved in disseminating information on misoprostol, it was felt that the survey would provide information to improve or redirect its work.

## 3 RESULTS

**Figure 1:**
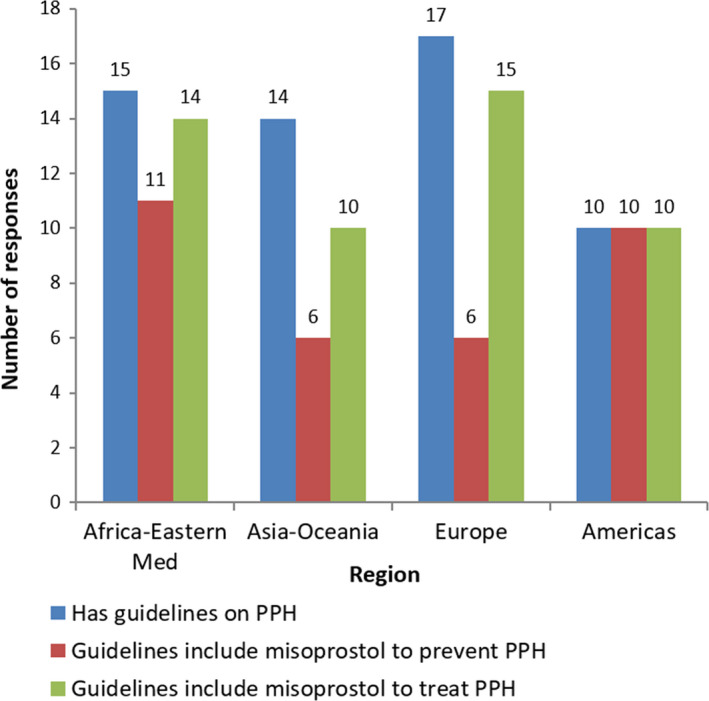
Existence and content of guidelines by region.

Completed surveys were received from 69 (53%) of the 130 MAs (Table 1). The survey found that many countries lacked complete, up‐to‐date, evidence‐based national guidelines and EMLs per inclusion of misoprostol for prevention and treatment of PPH. Of those responding to the survey, 56 (81%) reported that their country has national guidelines on PPH management. Of this number, 33 (59%) included recommendations for misoprostol for prevention of PPH, and 49 (88%) included recommendations for misoprostol for treatment of PPH (Fig. 1). Only 33 (59%) had recommendations for both indications. Similarly, of those responding to the survey, 42 (61%) reported that misoprostol was listed on their national EML. Of this number, 23 (55%) indicated its inclusion was listed for PPH prevention, 31 (74%) indicated its inclusion was listed for PPH treatment, and only 22 (52%) included it for both PPH indications.

**Table 1:** Responders by FIGO region

FIGO region	Total number of Member Associations	Responded No. (%)
African–Eastern Mediterranean	37	17 (46)
Asia–Oceania	26	17 (65)
Europe	44	24 (55)
Latin America	20	9 (45)
North America	3	2 (67)
Total	130	69 (53)

Regimens and conditions under which misoprostol was recommended varied greatly in national guidelines on PPH management. Eight different regimens were noted for prevention and 13 for treatment (Table 2). Conditions under which misoprostol is recommended for prevention of PPH included: for every delivery in any facility, for “high‐risk” deliveries, for deliveries outside facilities, when oxytocin is not available, and when an unskilled birth attendant is present at the delivery; some guidelines did not specify conditions for use. The same conditions were given in the case of misoprostol for treatment of PPH, with the additional condition “after failure of first line treatment with other agents.”

**Table 2:** Regimens and conditions recommended for misoprostol use (multiple responses possible)

Misoprostol for PPH prevention (n=33)		Misoprostol for PPH treatment (n=49)	
Regimen	No. (%)	Regimen	No. (%)
400 μg oral	3 (9.1)	400 μg oral	3 (6.1)
400 μg rectal/sublingual	1 (3.0)	400 μg sublingual/rectal	1 (2.0)
600 μg oral	19 (57.6)	400–600 μg rectal	1 (2.0)
600 μg rectal	4 (12.1)	600 μg oral	7 (14.3)
600 μg oral/rectal	1 (3.0)	600 μg rectal	4 (8.2)
800 μg sublingual	7 (21.2)	600 μg sublingual/rectal	1 (2.0)
800 μg rectal	3 (9.1)	600 or 800 μg rectal	1 (2.0)
1000 μg rectal	4 (12.1)	600–800 μg sublingual/rectal	1 (2.0)
Not specified	3 (9.1)	800 μg sublingual	17 (34.7)
		800 μg rectal	5 (10.2)
		800–1000 μg rectal/oral/sublingual	1 (2.0)
		800–1000 μg rectal	1 (2.0)
		1000 μg rectal	18 (36.7)
		Not specified	1 (2.0)

In response to the question about which international, regional, or other guidelines were used as principle referencing materials when drafting national guidelines, the most frequently cited were the WHO's recommendations for the prevention and treatment of postpartum hemorrhage, from 2012,^6^ followed by FIGO's guidelines for prevention and treatment of postpartum hemorrhage in low‐resource settings, from 2012,^8^ and the Royal College of Obstetricians and Gynaecologists’ (RCOG) guidelines on postpartum hemorrhage, prevention and management, from 2009.^9^ There is variation between the recommendations provided in these documents (Table 3), and national guidelines did not always resemble those that they had used for guidance. For example, for prevention of PPH, only 15 of the 46 responders (33%) who used WHO and/or FIGO guidelines as reference documents actually recommend the same regimen as WHO/FIGO (600 μg given orally), and two of these also listed alternative regimens. Similarly, for treatment of PPH, only 15 of the 46 responders (33%) who used WHO/FIGO guidelines as reference documents actually recommend the same regimen as WHO/FIGO (800 μg given sublingually), and five of these also listed alternative regimens.

**Table 3:** Principle referencing guidelines used to develop national guidelines and their recommendations

Guidelines	Used^a^	Prevention		Treatment	
		Regimen	When	Regimen	When
WHO (2012)^6^	42 (75)	600 μg oral	In settings where oxytocin is unavailable	800 μg sublingual	If oxytocin is unavailable or if the bleeding does not respond to oxytocin
FIGO (2012)^8^	35 (63)	600 μg oral	In settings where oxytocin is unavailable	800 μg Sublingual	In settings where oxytocin is unavailable
RCOG (2009)^b^ ^9^	25 (45)	600 μg oral	In situations where no oxytocin is available or birth attendants’ facilities are limited (e.g. a home delivery)	1000 μg rectal	Where parenteral prostaglandins are not available or where there are contraindications to prostaglandin F2
ACOG (2006)^c^ (reaffirmed 2013)^19^	23 (41)	Not included	—	800–1000 μg rectal	Not specified
FLASOG (2012)^d^ ^20^	8 (14)	600–800 μg rectal	—	800 μg sublingual	Not specified

a Used means listed in the survey as one of the principle referencing guidelines when drafting national guidelines; values are given as number (percentage).

b In December 2016, RCOG published its second edition guideline on the prevention and management of PPH. The revised guideline now recommends 800 μg sublingual, instead of 1000 μg rectal administration, for the treatment of PPH. Recommendations for prevention have been removed.

c In October 2017, ACOG published a practice bulletin on PPH that replaced the 2006 bulletin. The bulletin recommends 600–1000 μg oral, sublingual, or rectal administration for treatment of PPH.

d The FLASOG 2012 guidelines were listed as part of the survey owing to an understanding that there was greater awareness of this guideline than the 2013 updated version.

In response to the question about which lead agencies were involved in the creation of national guidelines, the most often cited were the obstetrics and gynecology association (37/56, 66%) and Ministry of Health (34/56, 61%). The most current versions of the guidelines had been published between 2006 and 2016; 20 (36%) had a year scheduled for their review (ranging from 2016 to 2020), 10 (18%) were reported as “currently underway,” and 26 (46%) had no review planned or the timescale for review was not known. Of 55 responders, 53 (95%) reported that their association of obstetrics and gynecology would be involved in the next review of the guidelines, and 45 (80%) reported that minor revisions to the guidelines were possible between reviews. No follow‐up question was asked to identify any cases where minor revisions had been made between reviews.

**Figure 2:**
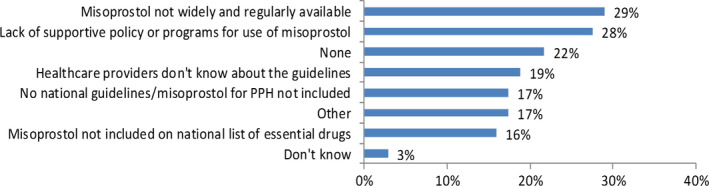
Challenges in implementing guidelines that include use of misoprostol for PPH.

The most common challenges to updating and implementing guidelines were: lack of supportive policy or programs for use of misoprostol; misoprostol not widely and regularly available; misoprostol not included on national EML; healthcare providers not knowing about the guidelines; lack of national guidelines/misoprostol not included in national guidelines; and misoprostol not being registered for use (this was not one of the listed potential challenges but was entered by responders under the option “Other”). Of the 69 responders, 15 (22%) reported no challenges (Fig. 2).

## 4 DISCUSSION

The findings of the present study support existing evidence that many countries either do not have national guidelines on PPH or that their guidelines are not sufficiently up‐to‐date.^10^, ^11^ It is vital that countries have up‐to‐date, comprehensive, and evidence‐based national guidelines on emergency obstetric care as evidence shows a direct relationship between guidelines, improved clinical practice, and better maternal outcomes. A recent systematic review evaluated the impact of new or updated guidelines for PPH management implemented in clinical practice. In four of seven trials included in the review, the numbers of PPH cases declined after the intervention, leading the authors to conclude that having guidelines for PPH management can have a positive impact on reducing the number of PPH cases.^7^ A similar study in Kazakhstan concluded that implementation of PPH management guidelines had a positive effect on PPH prevention, diagnostics, and management. The evidence suggested that guidelines led to a more deliberated approach to the treatment of PPH.^12^

Clinical guidelines must be disseminated in order to affect change; however, this survey identified healthcare provider lack of awareness of guidelines as a key challenge. It has been noted by others that clinical guidelines are often not disseminated to healthcare workers or are unclear and ambiguous, which makes them difficult to follow^13^, ^14^, ^15^; whereas guidelines that are easy to access and understand have a greater chance of being read and implemented.^16^

The finding that misoprostol was often absent from guidelines is of concern, especially in countries where a high number of women deliver in healthcare facilities in the community or at home, where access to oxytocin may be limited. Since guidelines on clinical practice also influence which drugs get added to national EMLs, what providers learn, and how they practice, it is imperative that these guidelines include misoprostol. It may be that current evidence on misoprostol is not reaching those tasked with writing guidelines or that evidence is unclear or incoherent. The survey showed that guidelines are often not revised regularly, which may be why they do not reflect current evidence. Further, in some countries there do not appear to be set procedures for uptake of new clinical evidence and guideline revision, or there are limited resources for making these happen.

The survey showed that while many MAs reported using WHO and/or FIGO guidelines as key referral documents, only approximately half then recommended the same regimen. While adaptations of international guidelines are acknowledged to be needed to make them locally appropriate and better able to meet the specific needs of each country and health service, WHO notes that: “Modifications to the recommendations, where necessary, should be limited to weak recommendations and justifications for any changes made in an explicit and transparent manner.”^6^ Given that modifications have been made to strong recommendations (i.e. those based on high‐quality evidence), and alternatives recommended are based on limited data and may be pharmacokinetically inferior,^17^ further work is needed to examine how international guidelines are used and what additional information determines the regimen that is selected nationally.

Differences between recommendations in international and regional guidelines may create an additional challenge for countries looking for external guidance when developing their own national protocols/guidelines. Additionally, it is interesting to note that some countries or regions tend to place closer attention and allegiance to specific external recommendations than others. For example, anecdotal evidence suggests MAs in Latin America look to FLASOG for guidance and many MAs in Africa look to the RCOG for guidance. This can be problematic as the RCOG guidelines do not look at African countries when formulating their recommendations, but to their own members—obstetricians and gynecologists in the UK. Therefore, low‐resource countries that are heavily reliant on guidelines from high‐resource countries may not be making the best recommendations for their particular country. They may instead focus on reinforcing higher technologies that may not be the most feasible or sustainable in lower‐resource settings. The RCOG and other high‐resource country guidelines may not need to include misoprostol at all owing to the common usage of oxytocin and the absence of barriers that would mean referencing alternatives. Obviously, the resource setting should determine the recommendation, which is why WHO suggests countries adapt international guidelines to their specific context. When we notified the RCOG of the high proportion of MAs using RCOG guidelines, we received the following response: “In their 2016 updated guidelines, RCOG removed misoprostol for prophylaxis as evidence suggested it was no better, and possibly not quite as good as, the current well‐established prophylaxis with syntocinon. The RCOG accepts, however, that misoprostol has an important role to play in settings where oxytocin is not readily available.”

A similar issue has occurred with the route of administration. The rectal route (how providers initially started using misoprostol) was often included in national guidelines, whereas it is not included in FIGO and WHO recommendations owing to its pharmacokinetic profile, which may not be associated with the best efficacy and because there is limited robust evidence on this route, while there is a plethora of evidence supporting the oral and sublingual routes for prevention and treatment. Follow‐up enquires were made after the survey with other obstetricians and gynecologists to understand why this route continued to be recommended. The reasons provided were that in many university hospitals the diagnosis of PPH and, therefore, the use of misoprostol may occur in women who are already being treated at a high level (i.e. they may be wearing an oxygen mask and be confused and disoriented, unconscious, or uncooperative) making the oral routes less favorable. In addition, for the obstetrician wearing surgical gloves these would have to be removed in order to put a tablet in the mouth, which also makes the rectal route favorable. Furthermore, the rectal route would be chosen during cesarean delivery. While these are valid reasons for using the rectal route, these circumstances are very different to what could be happening in a low‐resource setting where nonrectal routes should be used. This again implies that national guidelines may not be suitably tailored for low‐resource settings. In future, either international organizations should work together to synthesize evidence and potentially align their recommendations to ensure greater harmony with the aim of providing greater clarity for the development of national guidelines, or international bodies should work closer with national ones to look at regional needs and decide what is required in their particular setting.

In this survey, the most common challenges for implementing guidelines that include use of misoprostol for PPH were that misoprostol is not widely and regularly available and there is a lack of supportive policy and programs. This supports other findings that recognize that guidelines alone are insufficient in the absence of a supportive policy environment, without broad dissemination, scalable programs and training, and ensuring the availability of such commodities throughout all health facilities. A 2008 systematic meta‐review by Francke et al.^16^ concluded that effective strategies for influencing the implementation of clinical guidelines often have multiple components and that the use of one single strategy is less effective. This was also noted by Rizvi et al.^18^ who found that a revision of clinical guidelines on PPH together with dissemination to staff and use of practice drills led to a significant reduction in the incidence of massive PPH and a 100% adherence to the guidelines, which resulted in a significant reduction in maternal morbidity. Lack of familiarity with guideline content, lack of support from peers or superiors, and insufficient staff and time also impact implementation.^16^ Thus, those involved with guideline development must also be involved with strategies that ensure that their implementation is possible.

A limitation of the study was that the information received from MAs was not substantiated. The responses were based on the views and understanding of one senior person within the national society, and it may be possible that the respondent may not have had the most accurate or up‐to‐date information. The response also represented the view of one expert provider in the country and was not based on information from multiple providers. Additionally, what was reported may not reflect actual provider practices, which was beyond the scope of the survey.

Another limitation was that the survey was conducted in English, and while Spanish and French translations were provided upon request, this may have impacted on responses from non‐English speaking MAs. The survey also only focused on written national guidelines and, given wide access to the internet, it is unclear how many providers actually know about or rely on national documents versus information they obtain online using a range of sources. In this survey we did not ask if providers actually referred to their national guidelines or explore the use of other online resources such as videos and articles to guide clinical practice.

Further, the survey was limited in that it did not ask about which healthcare providers were able to give key medicines such as uterotonics, and was directed at the guidelines for obstetricians and gynecologists rather than other healthcare providers. It would also be interesting to examine guidelines provided for nonspecialist providers, such as midwives, who often attend deliveries, to see whether the same gaps and needs exist.

## 5 CONCLUSION AND RECOMMENDATIONS

In FIGO's guideline document on prevention and treatment of postpartum hemorrhage in low‐resource settings,^8^ FIGO calls professional associations to action—to work toward incorporation of recommendations into current guidelines, competencies, and curricula, and ensure that current best‐evidence regimens are adopted. FIGO reinforces this call to action and asks MAs without guidelines to initiate dialogue with the Ministry of Health to create new guidelines, and for MAs with guidelines that do not reflect new best evidence to advocate for timely revisions. Further, regional and international societies should prioritize clinical updates and dissemination of new guidance and practice to a range of provider cadres. Given the global shortage of specialist clinicians in low‐resource settings, there is a need to create a pathway to better guidance, mentoring, and information sharing of the best clinical evidence within an environment of task sharing. Thus, rather than look at guidelines for PPH management by cadre, what is needed is more explicit discussion of task sharing within guidelines for different cadres of healthcare providers.

It is hoped that the findings presented here can be used in collaboration with partners to offer assistance to countries that do not have guidelines or are revising national guidelines to ensure that they are comprehensive, evidence‐based, and appropriate for their setting. These findings can also be used with partners to offer assistance to countries that do not have misoprostol listed on their EMLs. They could also be useful for discussion with partners when revising international guidelines to raise issues of conformity, dissemination, and implementation. They will also be used to guide FIGO's work disseminating evidence on misoprostol and other promising technologies for the management of PPH in the future.

## AUTHOR CONTRIBUTIONS

Both authors contributed to the writing of the commentary. JLM led on the survey design, and coordinated the data collection. JLM conducted the primary data analysis, with SK conducting further analysis.

## CONFLICTS OF INTEREST

The authors have no conflicts of interest.
